# Immune Function in Critically Ill Septic Children

**DOI:** 10.3390/pathogens10101239

**Published:** 2021-09-25

**Authors:** Katherine Elizabeth Bline, Mark W. Hall

**Affiliations:** Division of Critical Care Medicine, Department of Pediatrics, Nationwide Children’s Hospital, Columbus, OH 43205, USA; Mark.Hall@nationwidechildrens.org

**Keywords:** sepsis, pediatric, inflammation, immune, modulation, suppression, response, TNFα

## Abstract

The inflammatory response in pediatric sepsis is highly dynamic and includes both pro- and anti-inflammatory elements that involve the innate and adaptive immune systems. While the pro-inflammatory response is responsible for the initial clinical signs and symptoms of sepsis, a concurrent compensatory anti-inflammatory response often results in an occult, but highly clinically relevant, form of acquired immunodeficiency. When severe, this is termed “immunoparalysis” and is associated with increased risks for nosocomial infection, prolonged organ dysfunction, and death. This review focuses on the pathophysiology and clinical implications of both over- and under-active immune function in septic children. Host-, disease-, and treatment-specific risk factors for immunoparalysis are reviewed along with immune phenotype-specific approaches for immunomodulation in pediatric sepsis which are currently the subject of clinical trials.

## 1. Introduction

Sepsis represents a dysregulated host response to infection that, when severe, results in organ dysfunction. Sepsis remains an important public health problem and significant cause of morbidity and mortality in children worldwide [1,2]. While the timely recognition of sepsis and provision of fluid resuscitation and antibiotics have resulted in improvements in sepsis outcomes over the last two decades [3,4], over 7000 children still die of sepsis every year in the United States alone [5]. The pathophysiology of the immune response in sepsis is an attractive target for the development of novel treatments to improve outcomes in critically ill children with sepsis. 

Historically, most studies of sepsis interventions have focused on supportive care (e.g., antimicrobials, intravenous fluids, and other bundle elements) *or* the use of anti-inflammatory therapies. Indeed, although sepsis is triggered by an initial infectious insult, much of the damage done to the host is not directly due to the pathogen but to the dysregulated host inflammatory response that can persist even after the initial infection has been controlled. We now know that the immune response in sepsis is highly dynamic, with many children demonstrating a compensatory *anti-inflammatory* response that represents a clinically important, occult form of acquired immune deficiency. Understanding the variability of the immune response in children with sepsis will allow the development of new, personalized approaches to sepsis treatment. 

## 2. Innate and Adaptive Immune Systems

**Figure 1:**
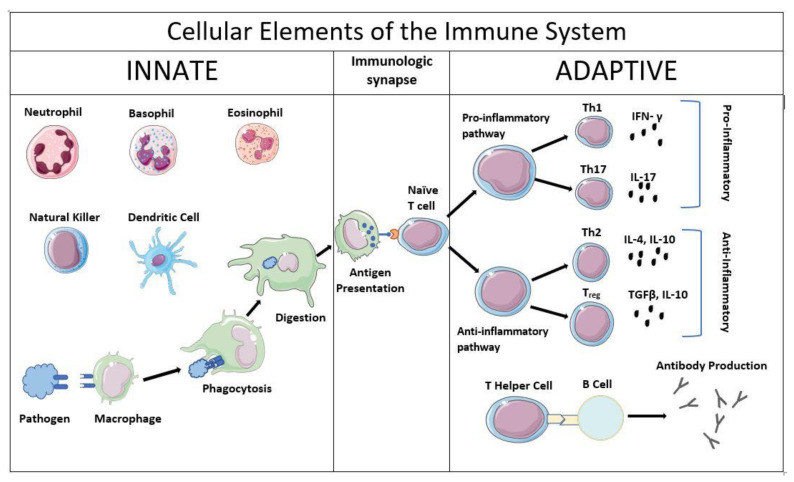
Cellular Elements of the Immune System. The immune system consists of two distinct but overlapping systems, the innate (primarily myeloid) and adaptive (lymphoid). The innate immune system provides the first line of defense against pathogens and the adaptive immune system is antigen-specific and provides longer lasting protection.

The immune system is comprised of two arms: the innate and adaptive immune systems (Figure 1). These two systems are comprised of immune cells and mediators that function to detect and remove harmful pathogens and to promote the remodeling of injured tissues. The innate immune system identifies broad classes of pathogen-associated molecular patterns (PAMPs) through constitutively expressed PAMP receptors, such as Toll-like receptors (TLRs) including the lipopolysaccharide (LPS) receptor TLR4 (6).

This is typically the first cellular arm of the immune system to respond to an infection. Activated innate immune cells, including neutrophils, monocytes, macrophages, and dendritic cells, initially produce proinflammatory chemokines and cytokines which make the local environment favorable for fighting infection and recruit other immune cells to the affected area. When this response spills over to the systemic circulation, however, the clinical findings of sepsis manifest. Examples of proinflammatory cytokines typically produced by innate immune cells include interleukin (IL)-1β and tumor necrosis factor (TNF)α. In addition to cytokine production, most innate immune monocytes are capable of phagocytosis of pathogens and intracellular killing. Antigenic peptides from the digested pathogen are then loaded onto class II major histocompatibility complex (MHC) molecules like human leukocyte antigen (HLA)-DR for presentation to the adaptive arm of the immune system.

The adaptive immune system is comprised of lymphocytes which, while often slower to respond to infection, are much more *specific* to a given pathogen. The adaptive immune system is also capable of generating long-lasting memory cells, which allows for a more rapid and efficient response to recurrent infection. Adaptive immune cells include T lymphocytes and B lymphocytes. T cells include CD8+ cytotoxic T cells which effect cellular killing through the release of lytic enzymes [6], and CD4+ helper T cells which produce cytokines that modify the activity of other immune cells and parenchymal cells. Helper T cells can differentiate from naïve CD4+ cells into one of multiple subgroups of helper T cells depending on the cytokine milieu in which they activated. While many of the helper T cell subtypes are beyond the scope of this review, the following subtypes merit discussion: Th1, Th2, Th17, and regulatory T cells (Tregs) [7]. Th1 cells produce proinflammatory mediators such as IL-2 and interferon (IFN)γ, while Th17 cells produce the even more potent proinflammatory cytokine IL-17. Th2 cells produce mediators characteristic of the anti-inflammatory and allergic responses including IL-4, IL-5, and IL-10, and are crucial in promoting B cell maturation and subsequent antibody production. Tregs are even more potently anti-inflammatory, producing IL-10 and transforming growth factor (TGF)-β. 

## 3. Two Sides of the Immune Response

**Figure 2:**
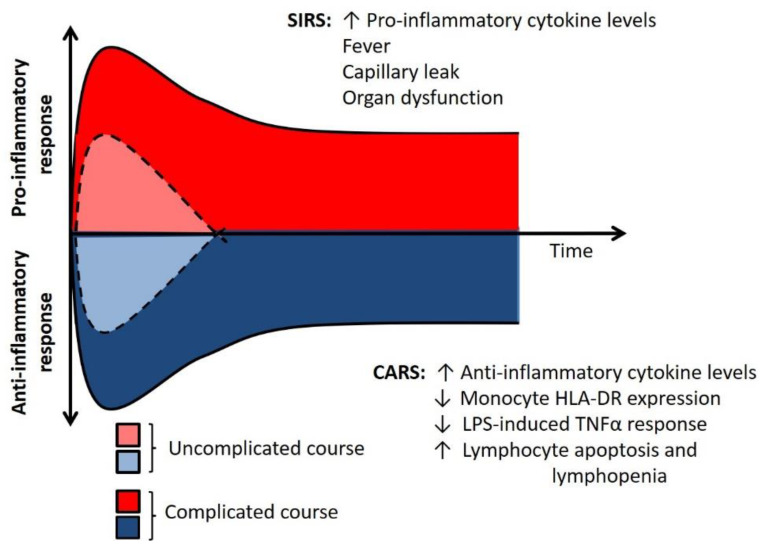
The dynamic immune response. Sepsis induces both pro-inflammatory and anti-inflammatory responses in the host. An exaggerated response of either, or both, processes is associated with increased risks for adverse outcomes. Adapted with permission from Hall et al., “Immunoparalysis in Pediatric Critical Care”, published in *Pediatric Clinics of North America* (2017).

Both arms of the immune system can produce mediators that are part of the Systemic Inflammatory Response Syndrome (SIRS). This is characterized by the production of proinflammatory cytokines such as TNFα, IL-1β, and IFNγ [8]. An uncontrolled, severe SIRS response can result in fever, capillary leak, organ dysfunction, and death [9] (Figure 2). Nearly concurrent with the initial SIRS response, the host also experiences a Compensatory Anti-Inflammatory Response Syndrome (CARS) that acts as a negative feedback mechanism to minimize inflammation-related damage. The CARS response is clinically occult but can be detected in the laboratory with specific testing. Findings consistent with the CARS response include decreased leukocyte cytokine production capacity; diminished expression of HLA-DR on the cell surface of circulating monocytes; systemic lymphopenia along with lymphocyte apoptosis in the spleen; and an increase in systemic levels of anti-inflammatory cytokines [10,11]. Notably, these increases in serum cytokines such as IL-10 often occur at the same time as elevations in pro-inflammatory biomarker levels, including IL-6 and IL-8. While the simultaneous elevation in systemic proinflammatory cytokines and reduction in leukocyte cytokine production capacity may seem paradoxical, it is important to understand that proinflammatory mediators are produced in abundance by injured parenchymal cells and stressed vascular endothelium. These may be the sources of systemic inflammation rather than circulating leukocytes in some cases.

## 4. Immunoparalysis

When severe, the CARS response is termed “immunoparalysis” and can be considered to be a clinically relevant form of acquired immune deficiency. Several diagnostic approaches have been used to define immunoparalysis. By far, the greatest volume of data on immunoparalysis in critically ill children with infection has come from measuring the ability of subjects’ whole blood to produce TNFα upon ex vivo stimulation with LPS. Blood samples from healthy children should produce TNFα robustly upon exposure to LPS. Children with immunoparalysis demonstrate marked reduction in this TNFα production capacity or “TNFα response”. In the most commonly used assay, a TNFα response < 200 pg/mL after stimulation of whole blood with 500 pg/mL of LPS for four hours has been repeatedly associated with mortality, increased nosocomial infection risk, and prolonged organ dysfunction in septic children [10,12,13,14,15]. In children with critical influenza infection, the addition of provocative testing of the antiviral retinoic acid–inducible gene-I (RIG-I) pathway may have added prognostic value compared to TLR4 pathway stimulation alone [15].

A reduction in monocyte HLA-DR expression such that < 30% of circulating monocytes are HLA-DR^+^ by flow cytometry has been associated with adverse outcomes in septic adults [16,17,18]. This has also been demonstrated in septic children [10], though lower degrees of suppression have also been associated with adverse outcomes [19]. This method of quantitation of HLA-DR expression carries a risk of bias due to lot-to-lot variability in fluorochromes and variations in cytometer settings. An alternative flow cytometric method involves the use of standard beads which permit the quantitation of the average number of HLA-DR molecules per cell. Adult data suggest that < 8000 HLA-DR molecules per monocyte is diagnostic of immunoparalysis [20]. Failure to improve monocyte HLA-DR expression over time has been more strongly associated with adverse outcomes in septic children rather than a discrete molecules/cell cutoff [21,22], though additional studies are needed.

Adaptive immune failure is also characteristic of immunoparalysis. An absolute lymphocyte count < 1000 cells/mm^3^ for seven days has been associated with increased odds of nosocomial infection and death in septic children with multiple organ dysfunction syndrome [23]. Similarly, both circulating lymphopenia and impairment of the ability of isolated CD4+ T cells to produce IFNγ in response to ex vivo stimulation with the lymphocyte stimulant phytohemagglutinin have been associated with infectious complications in septic children [24]. 

## 5. Gene Expression

Variability in the host immune response to infection includes variation in leukocyte gene expression. Multi-center whole-blood transcriptomic data have shown that suppression of mRNA expression for genes that encode lymphocyte signaling and glucocorticoid receptor pathway elements is associated with increased risk for mortality or prolonged MODS in children with septic shock [25]. This line of research has also suggested that response to sepsis treatment could vary by transcriptomic profile, or endotype. Specifically, septic children with reduced lymphocyte gene expression that were treated with hydrocortisone have been shown to have a higher mortality than subjects with more robust gene expression who received hydrocortisone treatment [25,26]. This is in agreement with innate immune function data that suggest that hydrocortisone treatment is associated with a longer duration of MODS in septic children with reduced TNFα response [27].

Genetic polymorphisms affecting gene transcription or resultant protein function, or epigenetic changes that modify gene expression have both been associated with alterations in the host inflammatory response. Examples of these are shown in Table 1. 

Epigenetic regulation of the inflammatory response is of particular interest in sepsis. Epigenetic changes influence how DNA is processed, and these changes are reversible without altering the underlying DNA sequence. Epigenetic mechanisms include histone modification, DNA methylation, and regulatory RNAs. Many human pathogens, such as viruses, bacteria, and fungi, enhance their own survival through epigenetic mechanisms that modulate the host response to infection [28,29]. Epigenetic regulation of immunological pathways has been shown to affect the differentiation of macrophages from monocytes, TNFα transcription, and expression of antigen-presenting genes, resulting in changes in immune activation and immunologic tolerance [30,31,32]. Studies of genomic and epigenetic influences on the host immune response have the potential to identify new approaches to sepsis risk stratification and to identify new therapeutic targets [33].

## 6. Immunothrombosis

The inflammatory response impacts the homeostasis of other host systems as well. The coagulation and immune systems are closely intertwined with a great deal of interaction between leukocytes and the endothelium that results in activation of the coagulation cascade. For example, apoptotic immune cells release damage-associated molecular patterns (DAMPs) including free DNA, histones, and other nuclear proteins. DAMPs can active other innate immune cells as well as the coagulation cascade [34]. In addition, when neutrophils are activated in response to a pathogen, they release neutrophil extracellular traps (NETs). NETs often contain arginine-rich histones including H3 and H4 which produce a hypercoagulable state through inhibition of fibrinolytic activity. NETs are also able to trap platelets, which can lead to severe thrombocytopenia. Higher concentrations of circulating NETs are associated with increased risk of disseminated intravascular coagulopathy (DIC), a condition that carries a higher risk of mortality [35]. Heparin is often used in the treatment of DIC to prevent thrombosis, and there has been greater focus on the immunomodulatory effects of heparin. In addition to being a thrombolytic, heparin has anti-inflammatory properties including inhibition of neutrophil function [36]. This interplay between the innate immune system and the microvasculature, termed “immunothrombosis” and associated treatments, is a growing area of interest in sepsis research.

## 7. Developmental Effects on Immune Response

Although severe sepsis is harmful in all children, younger age is an important risk factor, with septic newborns having a particularly high mortality rate of up to 1 per 1000 live births in the United States [37]. In a pediatric septic shock cohort, Wynn et al. demonstrated age-specific variations of the host response in the neonates compared to older infants, toddler, and school-aged children. Compared to the older children, the neonatal subjects had decreased expression of genes representing both the innate and adaptive immune pathways [38]. Others have demonstrated that cytokine profiles in neonates, both during healthy states and pathologic conditions, are different from those seen in older children and adults, with infants often having higher concentrations of circulating pro-inflammatory cytokines [39,40]. Data also suggest that the neonatal response to sepsis may rely more on innate immune function, demonstrated by upregulation of neutrophils and monocytes, accompanied by a decrease in T and B cells [41]. Further investigations stratifying the transcriptomic response and biomarkers by age have the potential to identify novel prognostic markers and therapeutic opportunities in young infants. 

## 8. Risk Factors for Immunoparalysis

### 8.1. Host Specific

In addition to the effects of developmental stage on the immune response [42], there are also sex-based differences that contribute to immune function [43,44]. For example, female adults have a stronger predisposition to autoimmune diseases and data suggest that expression of IFNγ may be regulated by estrogen levels [45]. As previously discussed, there are numerous genetic and epigenetic variations that are associated with alterations in the inflammatory response. The impact of these, and other, variants on pediatric sepsis outcomes, however, is poorly understood. An example is Neisseria meningitidis, which remains a leading causes of meningitis worldwide and has been linked to multiple single-gene mutations and polymorphisms associated with increased risk of susceptibility and disease severity [46]. A recent genetic analysis of septic adults with severe inflammation suggested a very high prevalence of polymorphisms in genes implicated in inborn errors of immunity and pathologic inflammation [47]. Multiple investigators are currently studying the genetics of pediatric sepsis, with a goal of identifying patients who may be at particularly high risk or who may benefit from gene-specific, personalized immunomodulation. 

### 8.2. Infection Specific

The initial infectious insult in patients with sepsis may represent an important factor in determining the overall host immune response. Human Immunodeficiency Virus (HIV), which directly targets T cells, is an example of a virus that has long been recognized as a significant cause of immune failure. Other more common infections, such as those caused by the influenza virus and *Staphylococcus aureus*, are also able to induce a state of immune suppression in the host through the induction of anti-inflammatory mediators or through the production of leukotoxins [12,48]. Similarly, infection caused by *Leishmania* parasites is known to induce significant host immune suppression through upregulation of IL-10 production [49]. 

### 8.3. Treatment Specific

Intended *and* unintended immunomodulation are common effects of treatments in the pediatric intensive care unit (see Table 2). Children with cancer, autoimmune diseases, and transplant recipients frequently receive immunosuppressive medications such as glucocorticoids, calcineurin inhibitors, and/or antibody-based therapies that target immune cells or pathways. While these are commonly stopped or tapered in the setting of sepsis, the practitioner may be compelled to continue them in the setting of uncontrolled graft rejection or rapidly progressive malignancy. Immunostimulatory therapies are increasingly being used in the context of clinical trials for the reversal of immunoparalysis in septic adults and children. Drugs currently under investigation for this purpose include recombinant granulocyte macrophage colony-stimulating factor (GM-CSF) (NCT03769844) and recombinant IFNγ (NCT04990232). Targeted anti-inflammatory therapy with drugs such as recombinant IL-1 receptor antagonist (anakinra) in subjects with hyperinflammation *without* immunoparalysis is also being evaluated in clinical trials (NCT04990232).

Many medications used in the pediatric ICU have unintended, often immunosuppressive, effects. For example, opioids have an immunosuppressant effect through the induction of TGF-β, and apoptosis of macrophages and lymphocytes. Furosemide is similarly associated with cytotoxic effects and immunosuppressive effects on monocytes [50,51]. Additional examples are provided in Table 2. It is critical that we develop an understanding of the immunologic effects of ICU pharmacopeia so that we can develop immune-friendly treatment regimens that promote restoration of normal immune function and avoid combinations of medications that promote inflammation or immunoparalysis.

## 9. Current Research and Future Directions

The development of personalized medicine-themed approaches to sepsis care includes the creation of immunophenotype-specific interventions that are designed to restore immunologic balance in the host. As noted above, the treatment of pediatric sepsis-induced immunoparalysis with drugs like GM-CSF is being evaluated in multi-center clinical trials. Others are evaluating drugs like recombinant IL-7 or anti-PD-L1 therapy in septic patients with lymphopenia [52,53]. It is also quite likely that *some* patients may benefit from targeted anti-inflammatory therapy, as has been shown in children with cytokine release syndrome following cellular therapies for malignancy. The key principle underlying all of these approaches is the development and use of prospective immune phenotyping approaches.

The heterogeneity of the host immune response to sepsis is attributable to both intrinsic and extrinsic factors, presenting a challenging treatment dilemma. It is highly unlikely that a single sepsis therapeutic targeting the inflammatory response will be effective in all patients. This is highlighted by the failure of clinical trials of anti-cytokine therapies in adults in the over the last 20 years in which the immunomodulator was given to subjects without a priori immune phenotyping (reviewed in [54]). In providing immunomodulatory therapies, it will be critical to ensure that the right drug is given at the right time to the right patient. This will require clinical trials of real-time, personalized, immune phenotype-directed immune modulation. Ongoing research in the areas of genomics, transcriptomics, and mechanisms of immunoparalysis has the potential to greatly inform the design of these clinical trials through subject selection, stratification, and the development of novel therapeutic approaches. By avoiding undesirable immunomodulation, promoting immune-friendly care, and developing immune phenotype-driven treatment approaches, we have the opportunity to improve outcomes in septic children around the world.

## Figures and Tables

**Table 1:** Genetic polymorphism and downstream effect.

**Genetic Polymorphism**	**Downstream Effect**
TNFα promoter (G→A at nucleotide -308)	Increased risk of sepsis among trauma patients
TLR4 (A→G at nucleotide +896)	Reduced LPS responsiveness, increased risk to Gram-negative sepsis
MASP2 deficiency	Decreased mannose-binding lectin protein, higher risk of sepsis and fatal outcome
BPI Taq gene	Predictor of sepsis severity in children in PICU
SNP in TLR1 at position -7202	Increased cytokine response and higher risk of mortality in sepsis
**Epigenetic Variant**	**Downstream Effect**
Dimethylation of histone 3 at lysine residue 9	Decreased macrophage response to LPS challenge
Histone methylation at promoter region of IL-12 gene	Decreased IL-12 protein production by dendritic cells in response to TLR stimulus, promote Th2 response
Acetylation of histone 3 and 4 proximal to IFNγ promoter	Increased differentiation of naïve T cells to Th1 cells
Decreased histone acetylation of Foxp3 gene	Upregulated expression of T regulatory cells

**Table 2:** Examples of overt and occult immunomodulatory effects of drugs in the pediatric ICU.

Drug(s)	Mechanism
**Overt Immunomodulation**	
***Suppression of the immune response***	
Tacrolimus, cyclosporine	Calcineurin inhibition-induced lymphocyte suppression
Sirolimus, everolimus	mTOR inhibition-induced lymphocyte suppression
Glucocorticoids	Lymphocyte apoptosis, suppression of pro-inflammatory gene transcription
Myeloablative chemotherapy	Bone marrow suppression
Anti-leukocyte monoclonal antibodies (e.g., rituximab and anti-thymocyte globulin)	Depletion of specific immune cell populations
Anti-cytokine agents (e.g., tocilizumab and anakinra)	Pathway-specific inhibition of inflammatory pathways
***Enhancement of the immune response***	
Granulocyte colony-stimulating factor	Increased numbers and function of neutrophils
Granulocyte macrophage colony-stimulating factor	Increased numbers and function of antigen-presenting cells (e.g., monocytes and dendritic cells)
Interferon-γ	Activation of innate and adaptive immune cells
**Occult Immunomodulation**	
***Suppression of the immune response***	
Antimicrobials	Bone marrow suppression (e.g., β-lactams, sulfonamides, and ganciclovir)
Catecholamines	Stimulation of β adrenergic receptors
Furosemide	Inhibition of cytokine production
Insulin	Inhibition of cytokine production
Opioids	Leukocyte apoptosis, inhibition of cytokine production, induction of TGFβ
Barbiturates	Inhibition of neutrophil function
***Enhancement of the immune response***	
Antimicrobials	Release of PAMPs through pathogen lysis (Jarisch–Herxheimer reaction)
Catecholamines	Stimulation of α adrenergic receptors
